# Significant Associations of the Interplay Between Stress Related Genes With Alzheimer’s Disease

**DOI:** 10.1093/geroni/igab046.2422

**Published:** 2021-12-17

**Authors:** Anatoliy Yashin, Deqing Wu, Konstantin Arbeev, Olivia Bagley, Igor Akushevich, Matt Duan, Arseniy Yashkin, Svetlana Ukraintseva

**Affiliations:** 1 Duke University, Durham, North Carolina, United States; 2 Duke University, Morrisville, North Carolina, United States

## Abstract

The lack of efficient medication against Alzheimer’s disease (AD) is the most important problem for this health disorder today. One possible reason for this -- the implementing medical interventions “too late in the disease stage” – has been recently addressed in the initiative that defined the preclinical AD stage by measuring changes in preclinical AD biomarkers. According to this definition, beta amyloid (Aβ) is one of the key preclinical AD biomarkers. Experimental studies showed that Aβ results from proteolytic cleavage of APP by β- and γ-secretases. Production of β-secretase involves BACE1 gene, activated by cellular stress response. This suggest that AD might be initiated by cellular stressors and that multifactorial regulation of AD is likely to be driven by genes involved in cellular stress response. In this paper we investigate whether interplay between SNPs from the EIF2AK4 gene involved in sensing cellular stress signals and the APP gene dealing with Aβ production may be associated with AD in human data. For this, we evaluated association of the interactions of the pairs of SNPs from these genes with AD in the analysis of HRS data. We found that interactions between several SNPs have statistically significant associations with AD. The results of this analysis confirm that the interplay between gene served as a sensor of cellular stress and gene involved in production of preclinical AD biomarker in response to stress may influence human AD. This analysis illustrates an important step towards translation of the results of experimental AD studies to human applications.

